# COVID-19 Pneumonia in Patients on Chronic Hydroxychloroquine Therapy: Three Cases of COVID-19 Pneumonia

**DOI:** 10.1155/2020/8822753

**Published:** 2020-10-22

**Authors:** Awan K. Rahman, Adam G. Purdy, Peter T. Ender

**Affiliations:** ^1^Section of Pulmonary and Critical Care, St. Luke's University Health Network, Bethlehem, PA, USA; ^2^Section of Infectious Diseases, St. Luke's University Health Network, Bethlehem, PA, USA

## Abstract

Hydroxychloroquine has gained popularity as a potential preventative and treatment of COVID-19 pneumonia due to its in vitro activity against the virus. These three cases of COVID-19 pneumonia of varying severities occurred in the setting of chronic hydroxychloroquine use. These cases argue against the use of hydroxychloroquine as a preventative or therapeutic option for COVID-19 pneumonia.

## 1. Introduction

SARS-CoV-2 is a nonsegmented, positive-sense RNA virus identified as the cause of the coronavirus pandemic of 2019 (COVID-19) [1]. The virus was first described in Wuhan, China, in December of 2019. Since then, SARS-CoV-2 has spread rapidly, causing a global pandemic. COVID-19 involves a wide spectrum of disease including asymptomatic infection, mild respiratory illness, and life-threatening respiratory failure with development of immune dysregulation and cytokine release syndrome (CRS) [2–4]. Thus far, only remdesivir has been approved by the FDA under Emergency Use Authorization (EUA) to treat COVID-19 and prevent progression of illness. There are currently no proven preventative antivirals [1].

Since the start of the COVID-19 pandemic, hydroxychloroquine has gained popularity as a potential preventative approach and therapeutic option for COVID-19 pneumonia. Hydroxychloroquine has been shown to inhibit SARS-CoV-2 replication in vitro [5–7]. The antiviral activity of chloroquine and hydroxychloroquine stems from their ability to inhibit fusion of the virus with cell membranes [8]. Other proposed mechanisms of protection include inhibition of nucleic acid replication and glycosylation of viral proteins [8]. The in vivo activity of hydroxychloroquine against SARS-CoV-2 is unclear.

Several studies with a variety of methodologies have argued for and against the efficacy of hydroxychloroquine in the treatment of COVID-19 [5–7, 9, 10]. While awaiting the results of ongoing randomized trials using hydroxychloroquine for both the treatment and prevention of COVID-19 pneumonia, limited data are available to confirm efficacy.

Some have suggested that chronic hydroxychloroquine use in the setting of rheumatologic disease may be beneficial for the prevention of COVID-19 [11]. The following cases describe COVID-19 pneumonia of varying severities despite chronic hydroxychloroquine treatment.

## 2. Case 1

**Figure 1:**
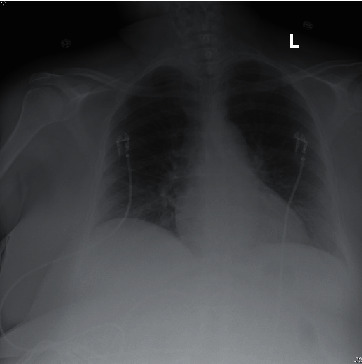
Normal radiograph without evidence of acute intrathoracic process.

A 55-year-old female with systemic lupus erythematous (SLE), fibromyalgia, type 2 diabetes, trigeminal neuralgia, and depression presented with a temperature of 102.4 F, myalgia, and shortness of breath. She tested positive for SARS-CoV-2 via polymerase chain reaction (PCR) from a nasopharyngeal swab and had elevated inflammatory markers on admission (Table 1). Her chest X-ray was clear (Figure 1). The patient had been maintained on hydroxychloroquine 200 mg twice daily and methylprednisolone 8 mg daily for treatment of SLE for years prior to becoming ill with COVID-19. The patient had an uncomplicated hospital course. She was continued on her outpatient regimen of hydroxychloroquine, and her methylprednisolone dose was increased to 16 mg daily on admission. Additionally, she was treated with vitamin D, vitamin C, zinc, and atorvastatin on presentation. Her symptoms and inflammatory markers rapidly improved. She did not require any supplemental oxygen. The patient was discharged after fifteen days in stable condition.

## 3. Case 2

**Figure 2:**
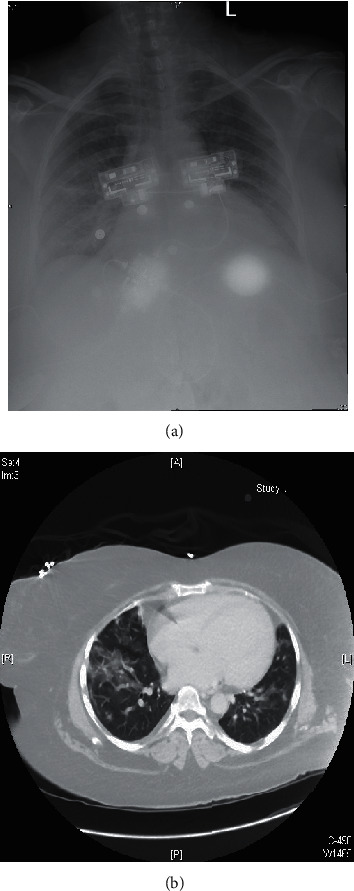
Chest X-ray demonstrating bilateral airspace opacities in the upper lung zones (a). CT chest demonstrating patchy ground-glass opacities (b).

A 42-year-old female with systolic and diastolic congestive heart failure with an ejection fraction of 20% secondary to ischemic cardiomyopathy, epilepsy, hypertension, bipolar disorder, and SLE presented with a temperature of 102.2 F, shortness of breath, and cough. She tested positive for SARS-CoV-2 via PCR of a nasopharyngeal swab on admission. She was chronically maintained on hydroxychloroquine 200 mg twice daily and prednisone 15 mg daily for SLE prior to this acute illness. While hospitalized, the patient had increasing oxygen requirements up to 15 L via nasal cannula and elevated inflammatory and coagulation markers (Table 1). Her chest X-ray revealed worsening airspace disease, and subsequent computed tomography (CT) of the chest revealed diffuse areas of ground-glass opacities and bibasilar consolidations (Figure 2). She was continued on her home hydroxychloroquine on admission, and her steroids were converted to intravenous methylprednisolone 80 mg daily on the fourth day of hospitalization. She was also treated with atorvastatin, vitamin C, and zinc on presentation. With these measures, she was eventually tapered off of supplemental oxygen and discharged to an inpatient rehabilitation unit on a prednisone taper after sixteen days of care.

## 4. Case 3

**Figure 3:**
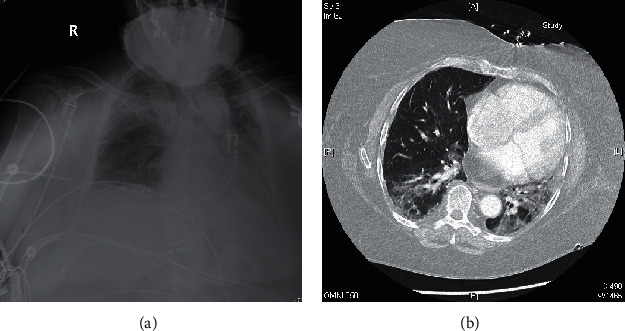
Chest X-ray demonstrating bilateral ground-glass opacities, worse in the right upper lung (a). CTA chest demonstrating peripheral, patchy ground-glass infiltrates, and consolidations, more prominent on the left (b).

A 62-year-old female with SLE, sarcoidosis, fibromyalgia, asthma, pulmonary fibrosis, and type 2 diabetes presented with a temperature of 102.8 F, cough, shortness of breath, fatigue, and diarrhea. She was in contact with a family member who had COVID-19 and subsequently tested positive for SARS-CoV-2 via PCR from a nasopharyngeal swab on admission. She had been chronically maintained on hydroxychloroquine 200 mg twice daily and methylprednisolone 12 mg daily for treatment of SLE and sarcoidosis prior to her current illness. The patient was continued on her outpatient dose of hydroxychloroquine, and her corticosteroid was converted to intravenous methylprednisolone 100 mg every 12 hours on the second day of hospitalization. She was also treated with vitamin D, vitamin C, zinc, and atorvastatin on admission. The patient clinically deteriorated despite these interventions. She was transferred to the intensive care unit for escalating oxygen requirements and developed increasing inflammatory and coagulation markers (Table 1) a few days after presentation. She received a one-time dose of intravenous tocilizumab on the fourth day of hospitalization. CT pulmonary angiogram was negative for a pulmonary embolism, but did reveal worsening bilateral ground-glass opacities (Figure 3). She was eventually intubated and mechanically ventilated. Additionally, the patient completed a five-day course of remdesivir starting on the tenth day of hospitalization. Unfortunately, she expired from respiratory failure nineteen days after admission.

## 5. Discussion

Until effective treatment options are available, the COVID-19 pandemic will continue to have a detrimental impact on healthcare and daily life. To expedite the availability of agents to treat COVID-19, repurposed medications are being clinically evaluated. Hydroxychloroquine has gained popularity as a potential treatment for COVID-19 because of its in vitro antiviral and immunomodulatory properties [6, 7, 12].

Published and pre-peer reviewed analyses of hydroxychloroquine in the treatment of COVID-19 have argued both for and against the benefit of this medication for the treatment of SARS-CoV-2 infection [5–7, 9, 10, 12]. These studies have methodologic limitations that hamper interpretation of the data. The lack of any consistent positive findings would argue against hydroxychloroquine being a potent and effective antiviral agent for the treatment of COVID-19.

Chloroquine, a close relative of hydroxychloroquine, has shown potent in vitro activity against influenza virus and other coronaviruses, including SARS-CoV-1. SARS-CoV-1 was the cause of the SARS epidemic in 2003 [13, 14]. In vivo studies using chloroquine in various animal models have failed to consistently translate into successful outcomes in treating these viral infections [3, 14]. In a randomized trial, chloroquine was ineffective at preventing influenza infection [15]. A comparison of chloroquine to hydroxychloroquine in vitro demonstrated that hydroxychloroquine was more effective against SAR-COV-2 [7]. This argues that hydroxychloroquine is a more potent agent than chloroquine for treating SARS-COV-2.

A more recent case series of individuals with rheumatic disease on hydroxychloroquine did not reveal any difference in the development of COVID-19 [16]. A randomized trial of postexposure prophylaxis using hydroxychloroquine did not reveal any protective effect of developing illness compatible with COVID-19 [17].

The presented cases are similar to the abovementioned case series and add further doubt to any potent efficacy for hydroxychloroquine as a prophylactic or therapeutic agent against COVID-19 pneumonia. In comparison, the presented cases further demonstrate that hydroxychloroquine use does not decrease the severity of illness. The included patients had varying severities of illness including critical illness requiring ventilator support. Chronic glucocorticoid treatment in these patients may have increased the risk of breakthrough infection in the setting of hydroxychloroquine, which may confound interpretation of these findings. Additionally, pre-existing conditions may have contributed to the severity of illness in these patients.

These three cases reveal breakthrough infection with SARS-CoV-2 despite the ongoing chronic use of hydroxychloroquine. They demonstrate variability in inflammatory and coagulation markers, as well as severity of clinical disease, despite all patients being on hydroxychloroquine and glucocorticoid therapy (Table 1). As the incidence of COVID-19 continues to increase, ongoing studies will offer insight into effective treatments for this devastating disease. The description of COVID-19 infection occurring despite chronic hydroxychloroquine use argues against this agent being a potent antiviral agent that will be effective in the prevention or treatment of this illness. Results of the ongoing prophylactic and treatment trials with this agent are eagerly awaited.

## Figures and Tables

**Table 1:** Peak markers of inflammation and coagulation.

	Case 1	Case 2	Case 3
D-dimer (*µ*g/ml)	—	2.28	6.27
Fibrinogen (mg/dL)	—	716	608
ESR (mm/hr)	13	38	78
CRP (mg/L)	173	162.3	280.5
Ferritin (ng/mL)	571	277	411
